# Increased C‐reactive protein concentration and suicidal behavior in people with psychiatric disorders: A systematic review and meta‐analysis

**DOI:** 10.1111/acps.13351

**Published:** 2021-08-25

**Authors:** Alessandro Miola, Veronica Dal Porto, Tal Tadmor, Giovanni Croatto, Paolo Scocco, Mirko Manchia, Andre F. Carvalho, Michael Maes, Eduard Vieta, Fabio Sambataro, Marco Solmi

**Affiliations:** ^1^ Neurosciences Department University of Padua Padua Italy; ^2^ ULSS 6 Euganea Psychiatry Department Padova Italy; ^3^ Unit of Psychiatry Department of Medical Sciences and Public Health University of Cagliari Cagliari Italy; ^4^ Unit of Clinical Psychiatry University Hospital Agency of Cagliari Cagliari Italy; ^5^ Department of Pharmacology Dalhousie University Halifax Nova Scotia Canada; ^6^ IMPACT ‐ the Institute for Mental and Physical Health and Clinical Translation School of Medicine Barwon Health Deakin University Geelong Australia; ^7^ Department of Psychiatry Faculty of Medicine King Chulalongkorn Memorial Hospital Chulalongkorn University Bangkok Thailand; ^8^ School of Medicine IMPACT Strategic Research Centre Deakin University Geelong Victoria Australia; ^9^ Bipolar and Depressive Disorders Unit Hospital Clinic Institute of Neuroscience University of Barcelona IDIBAPS CIBERSAM Barcelona Spain; ^10^ Department of Psychiatry University of Ottawa Ottawa Ontario Canada; ^11^ Department of Mental Health The Ottawa Hospital Ottawa Ontario Canada

**Keywords:** C‐reactive protein, inflammation, prevention, psychiatry, suicidal attempt, suicidal behavior, suicidal ideation, suicidality

## Abstract

**Objective:**

Suicide is a leading cause of death worldwide. Identifying factors associated with suicidality (suicidal ideation [SI]/suicidal behavior) could increase our understanding of the pathophysiological underpinnings of suicide and improve its prevention.

**Methods:**

We conducted a systematic review (PubMed/PsycInfo/Cochrane databases, up to September 2020) and random‐effect meta‐analysis including observational studies comparing peripheral C‐reactive protein (CRP) levels in suicidal versus non‐suicidal patients affected by any psychiatric disorder and healthy controls (HC). Primary outcome was the CRP standardized mean difference (SMD) between patients with high suicidality versus those with absent or low suicidality. Secondary outcomes were SMD of CRP levels between those with suicide attempt versus no suicide attempt, as well as between those with (high) versus low or absent SI. Quality of included studies was measured with Newcastle‐Ottawa scale.

**Results:**

Out of initial 550 references, 21 observational studies involving 7682 subjects (7445 with mood disorders or first‐episode psychosis, 237 HC) were included. A significant association of CRP levels with suicidality (SMD 0.688, 95% CI 0.476–0.9, *p* < 0.001) emerged. CRP levels were higher in individuals with high SI (SMD 1.145, 95% CI 0.273–2.018, *p* = 0.010) and in those with suicide attempt (SMD 0.549, 95%CI 0.363–0.735, *p* < 0.001) than non‐suicidal individuals (either patients or HC). Main analyses were confirmed in sensitivity analysis (removing HC), and after adjusting for publication bias. The cross‐sectional design of included studies, and the high heterogeneity of diagnosis and treatment limit the generalizability of these results. Median quality of included studies was high.

**Conclusion:**

CRP is associated with higher suicidality in patients with mental disorders. Large cohort studies longitudinally monitoring CRP levels are needed to explore its longitudinal association with suicidality.

**Summations**

- CRP levels are increased in patients affected by severe mental illness with elevated suicidality, after pooling data from 21 studies and 7682 subjects.
- Specifically, our study provides evidence of a large (SMD 1.145, 95% CI 0.273–2.018) association between CRP concentrations and suicidal ideation, as well as a medium (SMD 0.549, 95% CI 0.363–0.735) association with suicide attempt.

**Limitations**

- The observational and cross‐sectional design with small to moderate sample sizes of included studies, and the partial adjustment for clinical confounders limit the generalizability of these results.

## 1 INTRODUCTION

Suicide and suicide‐related behaviors are a public health problem. Each year, approximately 800 000 people die due to suicide, while it is estimated that the number of suicide attempts is over twenty times higher.^1^ Of those who die by suicide, over 90% suffer from a mental illness, most commonly major depressive disorder (MDD) in up to two‐thirds of patients.^2^, ^3^ Risk of suicide is particularly high among patients diagnosed with severe depression or bipolar disorder (BD), especially when there are concomitant mixed features or agitation, or co‐occurring substance use disorders.^4^ Indeed, BD carries a high suicide risk; it has been estimated that around 15–20% of patients with BD die by suicide.^5^, ^6^, ^7^ Moreover, suicidal behaviors (SBs) occur at a significantly greater rate in schizophrenia than in the general population, with a lifetime suicide rate in individuals with schizophrenia approximately of 10%.^8^

There are multiple factors and potential mechanisms that contribute to the complexity of SBs and suicidal risk in these mental disorders (depressive, BD, schizophrenia).^9^ Previous studies have focused on SB, suicidal ideation (SI), and non‐lethal suicidal attempt (SA) as a target. Such targets are promising for suicide‐related research since they strongly relate to a suicide outcome and are more frequent than suicide, with prevalence rates of 9.2% and 2.7% for SI and a non‐lethal SA, respectively.^10^ In addition, 29% of individuals with lifetime SI attempt suicide, suggesting that SI and SA map on a continuum of suicide risk.^10^, ^11^ Therefore, in order to address the high suicide rate worldwide, identification of contributors and markers of both SI and SA could be crucial in clinical practice.

The assessment of suicide risk based exclusively on the patient's clinical history has low specificity.^12^ In addition, previous reviews have examined the predictive validity of suicide assessment instruments, demonstrating poor performance in the prediction of subsequent suicide attempt and suicide.^13^, ^14^, ^15^, ^16^

A complex combination of psychosocial, biological, cultural, and environmental factors can result in SB.^17^, ^18^ While none of these factors can reliably predict suicide alone,^19^ suggestive evidence indicated that some biological markers are possibly related to increased SBs, including activation of immune‐inflammatory pathways.^20^, ^21^, ^22^, ^23^, ^24^ Two previous meta‐analyses revealed that inflammatory markers’ levels were increased in suicidal patients compared with healthy controls (HC), suggesting a possible important role of immune‐inflammatory pathways in SB pathogenesis.^20^, ^21^

More in detail, a possible pathway implicating immune‐inflammatory processes is through the release of cytokines and their consequences, which have neurotoxic effects, lead to a breakdown of the blood‐brain barrier, thereby allowing activated immune cells and their products to influence brain functions.^25^

C‐reactive protein (CRP), a positive acute phase protein, an inflammatory protein synthesized by Kupffer cells in the liver in response to increases in pro‐inflammatory cytokines including IL‐6, IL‐1, and tumor necrosis factor (TNF)‐α,^26^, ^27^, ^28^ is frequently employed in clinical and translational research given its detectability at lower levels using high‐sensitivity assays.^29^, ^30^ It is easily detected both in serum and in plasma samples,^31^, ^32^ hence widely used in clinical practice to measure the presence and severity of inflammation.^33^, ^34^

While activated immune‐inflammatory pathways, which imply CRP elevation, have been reported to significantly contribute to the pathophysiology (and incorporated in staging models) of mood disorders, including MDD and BD, as well as to the cognitive and symptomatic features of schizophrenia, and SBs,^20^, ^35^, ^36^, ^37^, ^38^ to date contradictory results emerged from studies focusing on CRP’s role in patients with SBs. While a few studies with relatively small sample size showed no significant associations between CRP and SB,^39^, ^40^, ^41^ other studies revealed statistically significant association in patients with mental disorders.^22^, ^42^, ^43^, ^44^, ^45^, ^46^ In some of these studies, increased CRP and pro‐inflammatory cytokines have been described in individuals following recent^43^, ^47^ and remote^42^ suicide attempts.

To the best of our knowledge, so far only one meta‐analysis pooled data on the association between CRP and suicidality, only focusing on people with depressive disorder.^48^ It remains unclear whether CRP is associated with suicidality beyond those with depressive disorder, whether the association is valid with both suicide ideation and SB, and to what extent.

## 2 AIMS OF THE STUDY

The aim of the present systematic review and meta‐analysis is to pool data from observational studies reporting on CRP and suicidality (ie, SB/SI), without any restrictions in terms of underlying diagnosis.

## 3 METHODS

### 3.1 Search

A systematic review was conducted by database inception, last search on September 3, 2020, via Ovid platform in PubMed/PsycInfo/Cochrane database. Search key was “(CRP OR C‐reactive protein OR hsCRP OR hs‐CRP) AND (suicide OR SB OR SA OR suicidal thoughts OR Self‐Mutilation OR suicide* OR SI OR commit suicide OR suicidality).” In addition, a manual search was conducted, screening the reference list of included trials and relevant reviews. Two authors independently (TT and VDP) screened the title and abstract of the initial list of articles and then collected the full text of potentially eligible ones, further assessing eligibility. The reason for the exclusion of studies was also recorded. If any disagreement emerged at this stage, it was solved by a third author (AM).

Observational studies comparing peripheral CRP concentrations between suicidal and non‐suicidal patients, and between suicidal patients and HCs were included, based on the following criteria: (1) Patients could have any psychiatric disorder defined according to standardized diagnostic criteria (ie, DSM, or ICD‐any version); (2) the study had to report on suicidality, defined as either SI, suicide attempt, or suicide; and (3) the study had to report CRP concentrations in peripheral sample. Excluded were studies not focusing on the patients with any psychiatric disorder and focusing on the general population, or not reporting CRP levels. We also excluded case reports, or case series, and reviews. We also excluded studies that compared CRP levels in subjects with medical disorders known to increase CRP levels. We did not apply any language restrictions.

### 3.2 Primary and secondary outcomes

The primary outcome was CRP concentration in subjects with suicidality (either SI or SB) versus those without or with lowest suicidality.

Secondary outcomes were CRP levels in those with suicide attempt versus no suicide attempt, as well as in those individuals displayed highest versus without or lowest SI.

### 3.3 Data extraction

Two authors (TT and VDP) independently extracted data with a third author (AM) solving persistent disagreements and making the final decision. The following variables were extracted into a predefined excel spreadsheet: author, year, country, underlying mental condition, population setting, age group, sample size, diagnosis, exclusion criteria, PCR concentrations, assay methods, outcomes, and funding.

Mean and standard deviation of baseline, endpoint, or change values of primary and secondary outcomes were extracted according to their availability in the published eligible studies. When such values were not reported in text or tables, but only in figures, data were extracted from figures via an online platform (https://automeris.io/WebPlotDigitizer/).

### 3.4 Quality assessment

Two authors (AM and VDP) independently assessed the quality of the included studies with the Newcastle‐Ottawa Scale (NOS), with an average score ≥7 (out of nine) indicating high quality.^49^

### 3.5 Statistical analyses

A PRISMA‐compliant^50^ systematic review and random‐effect^51^ meta‐analysis was conducted, when at least two studies reported the same outcome. When more than one outcome was reported in one study from the same sample, the mean of the effect sizes within each individual study was considered to avoid double counting and artificial narrowing of confidence intervals. Heterogeneity was assessed with the *I* ^2^ statistics for each analysis (with significant heterogeneity being indicated by *I* ^2^≥50%).^52^ Publication bias was assessed via Egger's test.^53^ We also calculated the fail‐safe number (estimated number of studies needed to move the effect size from significant to non‐significant), and trim and fill adjusted analysis^54^ in case of publication bias (namely Egger's test *p*‐value < 0.1). Because of different detection methods between studies, the standard mean difference (SMD) was computed as the effect size for the differences in CRP levels and calculated corresponding 95% confidence intervals. Analyses were run using comprehensive meta‐analysis v2.0 (CMA, version 2 ‐ meta‐analysis.com).^55^ Random‐effect meta‐regression was conducted to explore age, quality of included studies, and years of publications as potential moderators of the primary outcome. Sensitivity analyses were run removing HC to avoid confounding by indication and focusing on those studies comparing highest levels of SI versus lowest/no SI (secondary outcome only). Subgroup analyses were run to compare studies based on country, psychiatric condition affecting the population of interest, CRP type (high‐sensitivity, normal), CRP sampling (plasma vs. serum), and the quality of the studies.

## 4 RESULTS

### 4.1 Search results, characteristics, and quality of included studies

**FIGURE 1:**
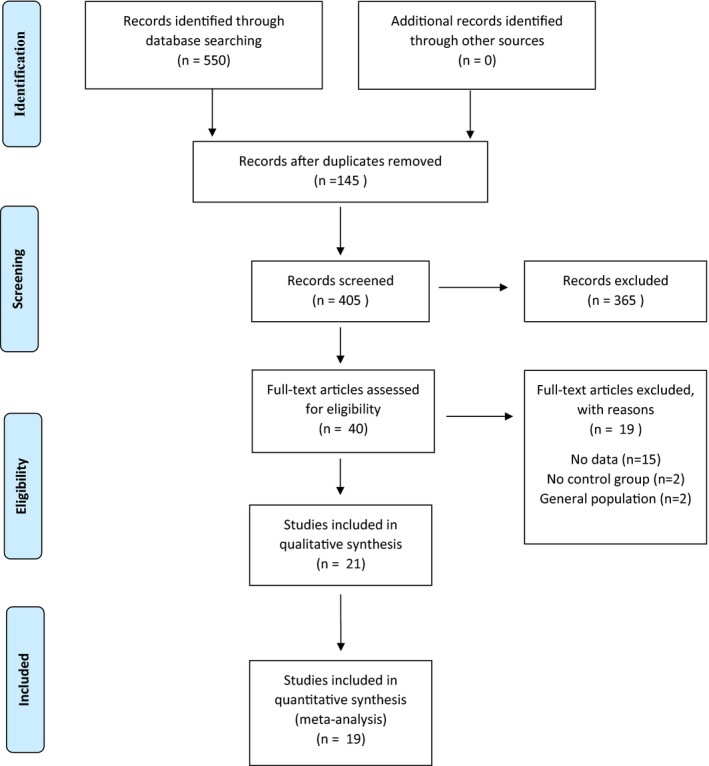
PRISMA

Search results and the study selection process are illustrated in Figure 1. Out of 550 initial hits, we screened 405 studies (after removing duplicates) at the title/abstract level, selecting 40 studies for full‐text assessment. We excluded 19 studies for specific reasons after full‐text assessment and ultimately included 21 studies. The complete list of the 19 studies excluded after full‐text assessment, with reasons for exclusion, is reported in Table S3 (Supplementary material, page 7). Detailed characteristics and references of included studies are reported in Table 1.

**TABLE 1:** Characteristics of included studies

Author, year	Design	Country	*N* (HC)	Age Mean	F%	MI diagnosis	Dg pop	Pop setting	Pop outcome	CRP type	Sample type	Assay method	Matching
Aguglia, 2019^62^	CC	Italy	632	49.68	27.37	DSM‐V	BD, LLSA,HLSA	Inpatient	SA	CRP	Serum	NA	Age, gender, marital/occupational status, and psychiatric diagnoses
Aguglia, 2020^63^	PC	Italy	432	49.15	77.06	DSM‐V	Mood	Inpatients	SA	NA	Serum	NA	NA
Al‐Amarei, 2019^25^	CC	Iraq	60 (30)	32.37	41.11	DSM‐IV	MDD	Inpatients	SA	CRP	Plasma	ELISA	NA
Cantarelli, 2014^60^	CS	Brazil	86	29.69	72.97	DSM IV	Mood	Inpatients	SA	CRP	Serum	immunoturbidimetric	NA
Chang, 2017^23^	CS	Taiwan	119	32.14	39.49	DSM IV	MDD	Outpatients	SI	hsCRP	Serum	IMMAGE 800 analyzer immunochemistry	NA
Courtet, 2015^42^	CS	France	600	39.78	72.17	DSM IV	MDD	Inpatients	SA	hsCRP	Serum	immunoturbidimetric	NA
De Berardis, 2013^61^	CS	Italy	30 (30)	25.9	56.6	DSM‐IV	FEP	NA	SI	NA	Serum	immunonephelometric	Age, Sex
Dolsen, 2020^11^	PC	Netherlands	2329	42.01	67.84	DSM‐IV	Mood	Mixed	SI,SA	hsCRP	Plasma	ELISA	NA
Ducasse, 2015^56^	CS	France	453	42.29	56.06	DSM IV	BD	Outpatients	SA	hsCRP	Serum	immunoturbidimetric	NA
Ekinci, 2017^24^	CC	Turkey	139(50)	42.69	69.41	DSM IV	MDD	Inpatients	SA	CRP	Serum	ELISA	Age, gender, education
Gambi, 2005^59^	RC	Italy	37	31.94	NA	DSM‐IV	MDD	Outpatients	SA	NA	Serum	immunonepehelometric	NA
Gibbs, 2016^43^	CC	USA	184	38.83	52.17	DSM IV	Mood, Psychosis	Inpatient	SA	hsCRP	Serum	NA	NA
Karlovic, 2012^39^	CS	Croatia	55(18)	NA	NA	DSM‐IV	MDD	Mixed	SA	NA	Serum	immunoturbidimetric assay	NA
Kim, 2019^9^	CS	USA	37	39.75	46.04	DSM‐IV	MDD	Inpatient	SA	CRP	Plasma	ELISA	NA
Loas, 2016^44^	CS	France	122	NA	NA	ICD−10	Mood	Inpatients	SA	CRP	Serum	immunoturbidimetry	NA
Odonovan, 2013^22^	CC	Ireland	74 (48)	49.16	70.99	DSM IV	MDD	Inpatients	SI	hsCRP	Plasma	immunonephelometry	NA
Oh, 2018^57^	CC	USA	1330	47.54	64.96	ICD−9	MDD	Mixed	SA	NA	NA	NA	NA
Peng, 2018^40^	CS	China	271	36.38	61	DSM IV	MDD	Outpatients	SA	hsCRP	Serum	Siemens Advia 2400 automatic biochemistry	NA
Priya, 2016^45^	CS	India	42 (42)	28.22	47.6	NA	Anxiety, MDD	Outpatients	SA	hsCRP	NA	ELISA	Age, gender
Vargas, 2013^41^	CS	Brazil	342	NA	66.05	DSM‐IV	Anxiety, MDD, Mood, Alcohol, Smoking, MS	Outpatients	SA	hsCRP	NA	immunonephelometry system	NA
Ventorp, 2015^58^	CS	Sweden	71 (19)	37.74	54.33	DSM‐IV	MDD	Inpatients	SA	NA	Plasma	ELISA	NA

Abbreviations: BD, bipolar disorder; CC, case‐control; CRP, C‐reactive protein; CS, cross‐sectional; DSM, Diagnostic and Statistical Manual; FEP, first‐episode psychosis; HLSA, high lethality suicide attempt; ICD, International Classification of Diseases; LLSA, low lethality suicide attempt; MDD, depressive disorder; MS, metabolic syndrome; NA, not available; PC, prospective cohort; RC, retrospective cohort; SA, suicide attempt; SI, suicidal ideation.

Overall, this meta‐analysis reports data from 7682 subjects (7445 affected by mental disorders, and 237 HC). Among persons with mental disorders, 453 were affected by BD, 2722 had a MDD, 2969 by a mood disorder, 30 had a first‐episode psychosis, and 1271 by mixed disorders. Age range was from 18 to 70 years old.

Twenty‐one studies included subjects with different disorders, one study included only subjects diagnosed with BD,^56^ ten studies investigated patients with MDDs,^9^, ^22^, ^23^, ^24^, ^25^, ^39^, ^40^, ^57^, ^58^, ^59^ and four studies included people affected by mood disorders.^11^, ^41^, ^44^, ^60^ Moreover, one study was focused on first‐episode psychosis,^61^ and five studies evaluated patients with mixed disorders.^42^, ^43^, ^45^, ^62^, ^63^

All continents except Africa and Oceania were represented. Specifically, four studies were conducted in Italy, three in the United States and in France, two in Brazil, one in Croatia, China, India, Iraq, Ireland, Sweden, Taiwan, and Turkey and one in The Netherlands. A detailed report on the quality of included studies according to the NOS scale is reported in Table 2.

**TABLE 2:** Quality of included case‐control and cohort studies, according to Newcastle‐Ottawa scale

Author, year	Selection				Comparability	Exposure (case‐control)/Outcome (cohort)			
	Case‐control studies								
	Case definition	Representativeness	Control selection	Control definition	Comparability	Ascertainment	Same ascertainment case‐control	No response rate	TOT
Aguglia, 2019^62^	1	1	0	1	2	1	1	1	8
Aguglia, 2020^63^	1	1	0	1	1	1	1	1	7
Al‐Amarei, 2019^25^	1	0	1	1	2	1	1	1	8
Cantarelli, 2014^60^	1	1	0	1	1	1	1	1	7
Chang, 2017^23^	1	1	0	1	1	1	1	0	6
Courtet, 2015^42^	1	1	0	1	1	1	1	1	7
De Berardis, 2013^61^	1	0	1	1	2	1	1	0	7
Dolsen, 2020^11^	1	1	0	1	2	1	1	1	8
Ducasse, 2015^56^	1	1	0	1	1	1	1	1	7
Ekinci, 2017^24^	1	1	0	1	2	1	1	1	8
Gambi, 2005^59^	1	1	0	0	1	1	1	0	5
Gibbs, 2016^43^	1	1	0	1	2	1	1	1	8
Karlovic, 2012^39^	1	0	1	1	2	1	1	1	8
Kim, 2019^9^	1	1	0	1	1	1	1	0	6
Loas, 2016^44^	1	0	1	1	1	1	1	0	6
Odonovan, 2013^22^	1	0	1	1	2	1	1	0	7
Oh, 2018^57^	1	1	0	1	1	1	1	1	7
Peng, 2018^40^	1	1	0	1	1	1	1	1	7
Priya, 2016^45^	1	0	1	1	1	1	1	1	7
Vargas, 2013^41^	1	1	0	1	1	1	1	0	6
Ventorp, 2015^58^	1	0	1	1	2	1	1	1	8

Case definition: 1 point if it is adequate, with independent validation. Representativeness of the cases: 1 point if there is a consecutive or obviously representative series of cases. Control selection: 1 point if there are community controls. Control definition: 1 point if they have no history of disease. Comparability of cases and controls on the basis of the design or analysis: 1 point if there are study controls for the most important factor or there are study controls for any additional factor. Ascertainment of exposure: 1 point if there is a secure record. Same ascertainment case‐control: 1 point if there is the same method of ascertainment for cases and controls. Non‐response rate: 1 point if there is the same rate for both groups.

The quality of included studies was high (NOS score ≥7) in 16 out of 21 studies, with a median score of 7.

### 4.2 Main and sensitivity analyses

Results of the main comparative random‐effects meta‐analysis, together with publication bias, sensitivity, and subgroup analyses are reported in detail in Table 3. Regarding primary outcome, CRP was higher in subjects with suicidality (either SB or ideation) versus those without or with lowest suicidality (*k* = 21, SMD 0.69, 95% CI 0.48 to 0.90, *p* < 0.001, *I* ^2^ 89.46%, medium effect size). Regarding secondary outcomes, CRP levels were elevated in those with suicide attempt versus those without suicide attempt (*k* = 17, SMD 0.55, 95%CI 0.36 to 0.73, *p* < 0.001, *I* ^2^ 85.68%, medium effect size), and the difference was even larger when focusing on SI (*k* = 5, SMD 1.14, 95% CI 0.273 to 2.02, *p* = 0.010, *I* ^2^ 95.43%, large effect size).

**TABLE 3:** Main, sensitivity, and subgroup meta‐analysis of primary and secondary outcomes

Comparison	Studies/samples/participants	SMD	95% CI	*p* value	*I* ^2^	Egger's test/fail‐safe number/trim and fill^a^
*Main analyses*						
Primary outcome						
Highest versus no/lowest suicidality	21/39/7682	0.688	0.476–0.9	<0.001	89.463	<0.001/1151/0.743, 95%CI 0.515–0.970
Secondary outcomes						
Suicide attempt versus no suicide attempt	17/30/6883	0.549	0.363–0.735	<0.001	85.683	0.002/706/0.674, 95%CI 0.471–0.878
Highest versus no/lowest suicidal ideation	5/9/2416	1.145	0.273–2.018	0.010	95.427	0.047/70/unchanged
*Sensitivity analyses (no confounding by indication – removing healthy controls)*						
Primary outcome						
Highest versus no/lowest suicidality	20/29/7445	0.688	0.459–0.917	<0.001	90.874	0.003/1036/0.787, 95%CI 0.538–1.035
Secondary outcome						
Suicide attempt versus no suicide attempt	16/23/6646	0.558	0.344–0.772	<0.001	89.207	0.064/638/0.805, 95%CI 0.534–1.078
Highest versus no/lowest suicidal ideation	5/6/2254	1.123	0.304–1.942	0.007	94.820	0.034/63/unchanged
*Subgroup analyses*						
By country						
Brazil	2/2/428	0.107	–0.182–0.397	0.468	0	Difference across subgroups *p* < 0.001
China	1/1/271	−0.009	–0.282–0.264	0.948	NA	
Croatia	1/6/28	0.821	–0.034–1.675	0.060	NA	
France	3/3/1900	0.280	0.138–0.422	<0.001	4.971	
India	1/1/84	0.866	0.419–1.313	<0.001	NA	
Iraq	1/2/56	2.195	1.520–2.870	<0.001	NA	
Ireland	1/3/81	2.096	1.531–2.662	<0.001	NA	
Italy	4/7/1225	1.004	0.455–1.554	<0.001	88.143	
Netherlands	1/2/2149	0.136	0.011–0.262	0.033	NA	
Sweden	1/4/90	0.823	0.367–1.278	<0.001	NA	
Taiwan	1/1/119	0.071	–0.289–0.431	0.699	NA	
Turkey	1/2/113	2.181	1.689–2.673	<0.001	NA	
USA	3/5/1738	0.335	0.187–0.483	<0.001	0	
By disorder						
Bipolar disorder	1/3/869	0.369	0.04–0.699	0.028	82.143	Difference across subgroups *p* < 0.001
Depressive disorder	10/21/2772	0.946	0.490–1.401	<0.001	93.281	
Mood disorders	4/4/2747	0.207	–0.024–0.438	0.079	46.287	
First‐episode psychosis	1/3/40	2.409	1.530–3.288	<0.001	NA	
Mixed disorders	5/8/1254	0.469	0.198–0.740	0.001	53.085	
By CRP/hs‐CRP						
CPR	12/25/3060	0.931	0.590–1.273	<0.001	89.772	Difference across subgroups *p* = 0.018
High sensitivity‐CPR/NA	9/14/4622	0.421	0.171–0.671	0.001	86.338	
By quality						
High quality	16/34/6825	0.756	0.508–1.005	<0.001	91.535	Difference across subgroups *p* = 0.189
Low quality	5/5/857	0.453	0.074–0.831	0.019	68.232	
By sampling						
NA	3/3/1856	0.405	0.067–0.743	0.019	68.920	Difference across subgroups *p* = 0.270
Plasma	5/12/2713	1.125	0.251–1.999	0.012	95.040	
Serum	13/24/3114	0.625	0.352–0.898	<0.001	88.188	

CI, confidence interval; CRP, C‐reactive protein; NA, not available; *I* ^2^, heterogeneity measure; SMD, standardized mean difference.

^a^ Measures of publication bias.

Sensitivity analyses removing HC to avoid confounding by indication substantially confirmed findings of main analyses.

### 4.3 Subgroup analyses

Detailed results are reported in Table 3. In subgroup analyses, effect sizes on primary outcome differed across countries. Specifically, Turkey had a large effect size and China a very small one.

In addition, the association of CRP levels with suicidality is confirmed among the different psychiatric disorders (BD; depressive disorder; mood disorders; first‐episode psychosis; and mixed disorders), suggesting the potentiality of CRP a transdiagnostic marker of suicide risk.

Moreover, the results are influenced also by the type of CRP detection (high sensitivity vs. normal). The association is confirmed with both techniques, yet the effect size is large with “regular” CRP array, but of medium magnitude with high‐sensitivity measurement.

Finally, no significant difference emerged from subgroup analyses regarding studies’ quality and sampling (plasma, serum).

### 4.4 Meta‐regression

Detailed results are reported in Table 4. Studies’ quality as a continuous measure, year of publication, and age of participants did not moderate results.

**TABLE 4:** Results of the meta‐regression

Moderator	*K*	Beta	95% CI	*p* value
Age	18	−0.021	−0.065 to 0.022	0.334
Quality	21	0.007	−0.277 to 0.430	0.671
Year	21	−0.007	−0.156 to 0.016	0.110

CI, confidence interval; *K*, studies; beta, moderator's effect size.

### 4.5 Publication bias

Detailed results are reported in Table 3. Potential publication bias was detected in all analyses, yet fail‐safe‐number ranged from 63 to 1151, and the significant nor the magnitude of effect sizes did not change substantially after trims‐and‐fill procedure.

## 5 DISCUSSION

This meta‐analysis shows a significant association of CRP levels with suicidality, and specifically a large association with SI, and medium association with suicide attempt pooling data from 21 studies and 7682 subjects (7445 with depressive disorders, BD, and schizophrenia spectrum disorders).

To the best of our knowledge, this is the first systematic review and meta‐analysis pooling data from patients with depressive disorder, BD, and schizophrenia spectrum disorder including first‐episode psychosis, and providing association estimates between CRP and SI and behavior separately.

Results are clinically relevant for several reasons. First, suicide has a high prevalence in the long‐time course of schizophrenia and affective disorders. Showing that CRP is associated with suicidality sums up to the established evidence of the association between a pro‐inflammatory status and depressive disorders,^48^, ^64^, ^65^ schizophrenia,^66^, ^67^, ^68^, ^69^ and BD.^70^, ^71^, ^72^ Findings of this evidence synthesis are consistent with previous reports suggesting possible pathogenetic pathways mediating CRP‐suicidality link.^21^, ^36^, ^73^ For instance, it has been shown that the microglia in the brain of patients died by suicide is activated, and that pro‐inflammatory mediators mRNA expression is increased in the prefrontal cortex of those dying by suicide, across different diagnoses.^21^, ^36^, ^73^ More precisely, levels of mRNA or proteins expression of inflammatory markers (IL1β, IL4, IL13, IL6, and TNF‐α) are higher in orbitofrontal cortex of suicide victims than in subjects died from other causes.^74^, ^75^ Also, pronounced microgliosis in specific brain regions (ie, dorsolateral prefrontal cortex, anterior cingulate cortex, and mediodorsal thalamus) of suicidal SMI patients has been observed.^73^, ^76^, ^77^ In addition, an increased level of inflammatory markers (CRP, IL2, IFN gamma, IL4, IL5, IL6, IL10, TNF‐α, IL6, IL8, and TGF‐β1) in the plasma and cerebrospinal fluid of suicidal patients has been described.^78^

Epidemiological evidence also points toward an association between immune‐system alterations and suicidality, as cohort studies have shown a link between allergy, atopic disorders, asthma, and suicidality.^79^, ^80^, ^81^ Converging evidence on the role of inflammatory mediators that increase suicidality also comes from evidence on the iatrogenic increased risk of SI with pro‐inflammatory cytokines used to treat hepatitis C or multiple sclerosis.^82^, ^83^

Environmental and interpersonal stressors (eg, social and familial threats, and negative life events) play a crucial role in triggering SB. These precipitating/contributing factors may significantly interact with predisposing factors that increase vulnerability to suicide according to a stress‐diathesis interplay.^84^ However, the main pathophysiological mechanisms underlying this link are still poorly understood.^78^ Stressful conditions can activate inflammatory signaling pathways in specific immune cells including monocytes and macrophages linked to abnormally enhanced pro‐inflammatory IL‐1β, IL‐6, and TNF‐α levels^85^, ^86^ on neuroglial cells. Pro‐inflammatory cytokines released by microglia may stimulate oligodendroglia releasing cytokines involved in myelination and astrocytes having phagocytic properties and secreting abnormally elevated cytokines.^87^, ^88^ Importantly, activated immune‐inflammatory pathways including elevated inflammatory cytokines and their multiple consequences have neurotoxic and excitotoxic effects on neurons leading to neurocognitive deficits associated with changes in neuroplasticity, synaptic sprouting, microglial sampling, lowered neurogenesis, changes in receptor expression and neuronal signaling, elevated nitro‐oxidative stress, and finally neurodegeneration.^89^ The state‐of‐the‐art theory of mood disorders and schizophrenia is that immune‐inflammatory neurotoxicity induces deficits in neuronal functioning which not only lead to depressive behaviors and psychosis but also SBs.^89^, ^90^, ^91^, ^92^

The routine analysis of serum CRP concentrations as a measure of inflammation and disease activity remains one of the most widely utilized assays in medicine. As a matter of fact, CRP is widely used as a marker of inflammation, infection and for risk stratification of cardiovascular events.^93^ As aforementioned, CRP is a relevant alternative for research, because of the short half‐life of other cytokines, and because the detectability at lower levels.^36^

Several studies have shown that CRP levels were significant associated with SI in patients with MDD,^22^, ^23^, ^24^ as well as in patients affected by generalized anxiety disorders.^94^ Such association seems to be specific for SI beyond the diagnostic group. For instance, in patients with depressive disorder, those with high levels of SI tend to show significantly higher levels of inflammatory cytokines (a composite score including TNF‐α, IL‐6, IL10, and CRP) compared to those with low levels of SI even after adjusting for depression severity.^22^, ^59^

Recently, Gan et al.^95^ found that several clinical features of BD including recent suicide attempt are associated with low‐grade inflammation (defined as CRP > 3 mg/L), after adjusting for BMI. In schizophrenia, in which a pro‐inflammatory status is present and is associated with clinical features,^96^, ^97^, ^98^, ^99^ higher suicide risk patients showed higher CRP levels than lower suicide risk patients and HCs.^61^

However, some contrasting findings can also be found in the literature. For instance, Karlovic and colleagues showed that IL6 was associated with SI within patients with depression with melancholic (32 patients) and atypical (23 patients) features, yet no difference was found in the concentration of CRP.^39^ In addition, CRP concentrations are increased also in suicide attempters with depressive disorder,^25^, ^58^ but no differences in the levels of serum hs‐CRP among suicide attempters and among non‐suicide attempters with MDD were found.^40^ Moreover, such association appears to be accounted for the presence of physical disease among patients receiving care in a medical setting.^57^

Conversely, a previous study showed a significant association between severe tobacco dependence and history of suicide attempt in BD patients, but not with level of CRP, independently of confounding factors.^56^

Finally, one study showed that CRP levels appeared not to be related to SB nor ideation in inpatients with schizophrenia who were retrospectively categorized according to CRP at admission (CRP > 1 vs. <1 mg/dl).^100^ Overall, this meta‐analysis is of importance in merging evidence from several studies with small sample size, generating a more credible effect size beyond small study bias and type II error in underpowered studies.

This meta‐analysis has several limitations. First, the majority of studies included in the present work adopted a cross‐sectional study design with small to moderate sample sizes, and therefore, we cannot draw firm conclusions on causality. Thus, it is unclear whether increased inflammation represents a state or trait associated with SB.

Second, studies in mood disorders should examine the association between SBs and a larger panel of immune‐inflammatory markers.^101^

Third, CRP levels are affected by clinical confounders including age, BMI, obesity, smoking, low serum vitamin D levels, low levels of physical activity, poor diet, allergies, childhood maltreatment, stress, sleep disorders, and subclinical infections.^102^, ^103^, ^104^, ^105^ For example, in BD, the increases in hs‐CRP are no longer significant after adjusting for the effects of BMI, age, and early lifetime trauma, which explain together 55% of the variance in hs‐CRP.^106^ Given that the present analysis included data published in original studies, we could not control for such confounding factors. Individual patient data meta‐analyses (IPDMA) could overcome such limitation.

Fourth, we were not able to control for ongoing treatment, despite the evidence indicates that treatment with antidepressants may affect cytokine and CRP levels.^107^, ^108^, ^109^, ^110^ IPDMA could overcome this limitation as well.

Fifth, it has been shown that the vast majority of evidence on biomarkers in the field of psychiatry is affected by several sources of bias. The present work is not exempt by different possible sources of bias, including reverse causality, small study effect, and excess of significance bias.^111^

Finally, we have not identified any study specifically focusing on schizophrenia, and the subgroup analyses by diagnosis remain exploratory, as just individual studies or too few studies have been conducted in each separate diagnostic group.

Future studies should assess more comprehensive profiles of the acute phase response (ie, changes in positive and negative acute phase proteins including haptoglobin, α1‐antitryprin, fibrinogen, sedimentation rate, and albumin), inflammation (acute phase proteins, IL‐1β, IL‐6, and TNF‐α, and complement factors), and cell‐mediated immune activation (M1 and Thelper‐1 cytokines).^37^ Since hs‐CRP is strongly influenced by BMI and age, future psychiatric research should always adjust for the effects of these confounders and publish the residualized data, which should be used in CMAs.

Also, specific meta‐research efforts primarily focusing on transdiagnosticity of CRP elevation as a marker or predictor of suicidality should be undertaken to test whether CRP is a transdiagnostic measure of suicidality.^112^, ^113^ Moreover, the interplay between brain‐immune markers and response to treatments, particularly in the field of suicide prevention, needs further studies.^114^, ^115^

In conclusion, we show that CRP is associated with risk of suicidality in patients with mental disorders, and in particular with SI.

## CONFLICT OF INTEREST

AM, VDP, TT, FS, GC, AFC, and MM have no conflict of interest to declare. MS has been a consultant for/received honoraria from Angelini, Lundbeck. MM has received honoraria by Angelini. EV has received grants and served as consultant, advisor or CME speaker for the following entities: AB‐Biotics, Abbott, AbbVie, Angelini, Boehringer‐Ingelheim, Dainippon Sumitomo Pharma, Ferrer, Gedeon Richter, GH Research, Janssen, Lundbeck, Novartis, Otsuka, Sage, Sanofi‐Aventis, Sunovion, and Takeda, outside the submitted work.

### PEER REVIEW

The peer review history for this article is available at https://publons.com/publon/10.1111/acps.13351.

## Supporting information

Supinfo S1

## Data Availability

The data that support the findings of this study are available from the corresponding author upon reasonable request.

## References

[1] WHO . Preventing suicide: A global imperative. WHO; 2021. Accessed February 7, 2021. http://www.who.int/mental_health/suicide‐prevention/world_report_2014/en/

[2] Mann JJ . Neurobiology of suicidal behaviour. Nat Rev Neurosci. 2003;4(10):819‐828. 10.1038/nrn1220 14523381

[3] Hawton K , Casañas i Comabella C , Haw C , Saunders K . Risk factors for suicide in individuals with depression: a systematic review. J Affect Disord. 2013;147(1–3):17‐28. 10.1016/j.jad.2013.01.004 23411024

[4] Baldessarini RJ . Epidemiology of suicide: recent developments. Epidemiol Psychiatr Sci. 2019;29:e71. 10.1017/S2045796019000672 31696818 PMC8061297

[5] Baldessarini RJ , Pompili M , Tondo L . Suicide in bipolar disorder: risks and management. CNS Spectr. 2006;11(6):465‐471. 10.1017/s1092852900014681 16816785

[6] Pompili M , Gonda X , Serafini G , et al. Epidemiology of suicide in bipolar disorders: a systematic review of the literature. Bipolar Disord. 2013;15(5):457‐490. 10.1111/bdi.12087 23755739

[7] Plans L , Barrot C , Nieto E , et al. Association between completed suicide and bipolar disorder: a systematic review of the literature. J Affect Disord. 2019;242:111‐122. 10.1016/j.jad.2018.08.054 30173059

[8] Sher L , Kahn RS . Suicide in schizophrenia: an educational overview. Medicina. 2019;55(7):361. 10.3390/medicina55070361 PMC6681260 31295938

[9] Kim DJ , Blossom SJ , Delgado PL , Carbajal JM , Cáceda R . Examination of pain threshold and neuropeptides in patients with acute suicide risk. Prog Neuropsychopharmacol Biol Psychiatry. 2019;95:109705. 10.1016/j.pnpbp.2019.109705 31326514 PMC7309511

[10] Nock MK , Borges G , Bromet EJ , et al. Cross‐national prevalence and risk factors for suicidal ideation, plans and attempts. Br J Psychiatry. 2008;192(2):98‐105. 10.1192/bjp.bp.107.040113 18245022 PMC2259024

[11] Dolsen MR , Prather A , Lamers F , Penninx B . Suicidal ideation and suicide attempts: associations with sleep duration, insomnia, and inflammation. Psychol Med. 2020:1‐10. 10.1017/S0033291720000860 32321599

[12] Lindh ÅU , Beckman K , Carlborg A , et al. Predicting suicide: a comparison between clinical suicide risk assessment and the Suicide Intent Scale. J Affect Disord. 2020;263:445‐449. 10.1016/j.jad.2019.11.131 31969276

[13] Bolton JM . Suicide risk assessment in the emergency department: out of the darkness. Depress Anxiety. 2015;32(2):73‐75. 10.1002/da.22320 25421638

[14] Bolton JM , Gunnell D , Turecki G . Suicide risk assessment and intervention in people with mental illness. BMJ. 2015;351:h4978 10.1136/bmj.h4978 26552947

[15] Larkin C , Di Blasi Z , Arensman E . Risk factors for repetition of self‐harm: a systematic review of prospective hospital‐based studies. PLoS One. 2014;9(1):e84282. 10.1371/journal.pone.0084282 24465400 PMC3896350

[16] Runeson B , Odeberg J , Pettersson A , Edbom T , Adamsson IJ , Waern M . Instruments for the assessment of suicide risk: a systematic review evaluating the certainty of the evidence. PLoS One. 2017;12(7):e0180292. 10.1371/journal.pone.0180292 28723978 PMC5517300

[17] Christodoulou C , Douzenis A , Papadopoulos FC , et al. Suicide and seasonality. Acta Psychiatr Scand. 2012;125(2):127‐146. 10.1111/j.1600-0447.2011.01750.x 21838741

[18] Rumble ME , Dickson D , McCall WV , et al. The relationship of person‐specific eveningness chronotype, greater seasonality, and less rhythmicity to suicidal behavior: a literature review. J Affect Disord. 2018;227:721‐730. 10.1016/j.jad.2017.11.078 29179142 PMC5805608

[19] Irigoyen M , Porras‐Segovia A , Galván L , et al. Predictors of re‐attempt in a cohort of suicide attempters: a survival analysis. J Affect Disord. 2019;247:20‐28. 10.1016/j.jad.2018.12.050 30640026

[20] Black C , Miller BJ . Meta‐analysis of cytokines and chemokines in suicidality: distinguishing suicidal versus nonsuicidal patients. Biol Psychiat. 2015;78(1):28‐37. 10.1016/j.biopsych.2014.10.014 25541493

[21] Ducasse D , Olié E , Guillaume S , Artéro S , Courtet P . A meta‐analysis of cytokines in suicidal behavior. Brain Behav Immun. 2015;46:203‐211. 10.1016/j.bbi.2015.02.004 25678163

[22] O'Donovan A , Rush G , Hoatam G , et al. Suicidal ideation is associated with elevated inflammation in patients with major depressive disorder. Depress Anxiety. 2013;30(4):307‐314. 10.1002/da.22087 23504697

[23] Chang C‐C , Tzeng N‐S , Kao Y‐C , Yeh C‐B , Chang H‐A . The relationships of current suicidal ideation with inflammatory markers and heart rate variability in unmedicated patients with major depressive disorder. Psychiatry Res. 2017;258:449‐456. 10.1016/j.psychres.2017.08.076 28886903

[24] Ekinci O , Ekinci A . The connections among suicidal behavior, lipid profile and low‐grade inflammation in patients with major depressive disorder: a specific relationship with the neutrophil‐to‐lymphocyte ratio. Nord J Psychiatry. 2017;71(8):574‐580. 10.1080/08039488.2017.1363285 28800269

[25] Al‐Amarei H , Rasheed S , Eidan A . C‐reactive protein and its relationship with lipid profile in suicidal and non suicidal adults with major depression. Indian J Public Health Res Dev. 2019;10:569. 10.5958/0976-5506.2019.01632.2

[26] Thompson D , Pepys MB , Wood SP . The physiological structure of human C‐reactive protein and its complex with phosphocholine. Structure. 1999;7(2):169‐177. 10.1016/S0969-2126(99)80023-9 10368284

[27] Cardiology RPM , Page P . C‐reactive protein: a simple test to help predict risk of heart attack and stroke. Circulation. 2003;108(12):e81‐85. 10.1161/01.CIR.0000093381.57779.67 14504253

[28] Maes M . A review on the acute phase response in major depression. Rev Neurosci. 1993;4(4):407‐416. 10.1515/revneuro.1993.4.4.407 7506108

[29] Black S , Kushner I , Samols D . C‐reactive Protein*. J Biol Chem. 2004;279(47):48487‐48490. 10.1074/jbc.R400025200 15337754

[30] Moutachakkir M , Lamrani Hanchi A , Baraou A , Boukhira A , Chellak S . Immunoanalytical characteristics of C‐reactive protein and high sensitivity C‐reactive protein. Ann Biol Clin. 2017;75(2):225‐229. 10.1684/abc.2017.1232 28377336

[31] Del Giudice M , Gangestad SW . Rethinking IL‐6 and CRP: why they are more than inflammatory biomarkers, and why it matters. Brain Behav Immun. 2018;70:61‐75. 10.1016/j.bbi.2018.02.013 29499302

[32] Sproston NR , Ashworth JJ . Role of C‐reactive protein at sites of inflammation and infection. Front Immunol. 2018;9:754. 10.3389/fimmu.2018.00754 29706967 PMC5908901

[33] Baumeister D , Akhtar R , Ciufolini S , Pariante CM , Mondelli V . Childhood trauma and adulthood inflammation: a meta‐analysis of peripheral C‐reactive protein, interleukin‐6 and tumour necrosis factor‐α. Mol Psychiatry. 2016;21(5):642‐649. 10.1038/mp.2015.67 26033244 PMC4564950

[34] Miller GE , Chen E , Parker KJ . Psychological stress in childhood and susceptibility to the chronic diseases of aging: moving toward a model of behavioral and biological mechanisms. Psychol Bull. 2011;137(6):959‐997. 10.1037/a0024768 21787044 PMC3202072

[35] Pandey GN , Rizavi HS , Zhang H , Bhaumik R , Ren X . Abnormal protein and mRNA expression of inflammatory cytokines in the prefrontal cortex of depressed individuals who died by suicide. J Psychiatry Neurosci. 2018;43(6):376‐385. 10.1503/jpn.170192 30371993 PMC6203549

[36] Courtet P , Giner L , Seneque M , Guillaume S , Olie E , Ducasse D . Neuroinflammation in suicide: toward a comprehensive model. World J Biol Psychiatry. 2016;17(8):564‐586. 10.3109/15622975.2015.1054879 26223957

[37] Maes M , Carvalho AF . The compensatory immune‐regulatory reflex system (CIRS) in depression and bipolar disorder. Mol Neurobiol. 2018;55(12):8885‐8903. 10.1007/s12035-018-1016-x 29611101

[38] Maes M , Sirivichayakul S , Matsumoto AK , et al. Increased levels of plasma tumor necrosis factor‐α mediate schizophrenia symptom dimensions and neurocognitive impairments and are inversely associated with natural IgM directed to malondialdehyde and paraoxonase 1 activity. Mol Neurobiol. 2020;57(5):2333‐2345. 10.1007/s12035-020-01882-w 32040834

[39] Karlović D , Serretti A , Vrkić N , Martinac M , Marčinko D . Serum concentrations of CRP, IL‐6, TNF‐α and cortisol in major depressive disorder with melancholic or atypical features. Psychiatry Res. 2012;198(1):74‐80. 10.1016/j.psychres.2011.12.007 22386567

[40] Peng R , Dai W , Li Y . Low serum free thyroxine level is correlated with lipid profile in depressive patients with suicide attempt. Psychiatry Res. 2018;266:111‐115. 10.1016/j.psychres.2018.05.059 29859497

[41] Odebrecht Vargas H , Vargas Nunes SO , Pizzo de Castro M , et al. Oxidative stress and lowered total antioxidant status are associated with a history of suicide attempts. J Affect Disord. 2013;150(3):923‐930. 10.1016/j.jad.2013.05.016 23856278

[42] Courtet PH , Jaussent I , Genty C , et al. Increased CRP levels may be a trait marker of suicidal attempt. Eur Neuropsychopharmacol. 2015;25(10):1824‐1831. 10.1016/j.euroneuro.2015.05.003 26032768

[43] Gibbs HM , Davis L , Han X , Clothier J , Eads LA , Cáceda R . Association between C‐reactive protein and suicidal behavior in an adult inpatient population. J Psychiatr Res. 2016;79:28‐33. 10.1016/j.jpsychires.2016.04.002 27135541

[44] Loas G , Dalleau E , Lecointe H , Yon V . Relationships between anhedonia, alexithymia, impulsivity, suicidal ideation, recent suicide attempt, C‐reactive protein and serum lipid levels among 122 inpatients with mood or anxious disorders. Psychiatry Res. 2016;246:296‐302 10.1016/j.psychres.2016.09.056 27744231

[45] Priya PK , Rajappa M , Kattimani S , Mohanraj PS , Revathy G . Association of neurotrophins, inflammation and stress with suicide risk in young adults. Clin Chim Acta. 2016;457:41‐45. 10.1016/j.cca.2016.03.019 27034054

[46] Cáceda R , Griffin WST , Delgado PL . A probe in the connection between inflammation, cognition and suicide. J Psychopharmacol. 2018;32(4):482‐488. 10.1177/0269881118764022 29552947 PMC9230995

[47] Ganança L , Oquendo MA , Tyrka AR , Cisneros‐Trujillo S , Mann JJ , Sublette ME . The role of cytokines in the pathophysiology of suicidal behavior. Psychoneuroendocrinology. 2016;63:296‐310. 10.1016/j.psyneuen.2015.10.008 26546783 PMC4910882

[48] Chen X , Pu J , Liu Y , et al. Increased C‐reactive protein concentrations were associated with suicidal behavior in patients with depressive disorders: a meta‐analysis. Psychiatry Res. 2020;292:113320. 10.1016/j.psychres.2020.113320 32717709

[49] Wells G , Shea B , O’Connell D , et al. The Newcastle–Ottawa Scale (NOS) for Assessing the Quality of Non‐Randomized Studies in Meta‐Analysis. 2000.

[50] Liberati A , Altman DG , Tetzlaff J , et al. The PRISMA statement for reporting systematic reviews and meta‐analyses of studies that evaluate health care interventions: explanation and elaboration. PLoS Medicine. 2009;6(7):e1000100. 10.1371/journal.pmed.1000100 19621070 PMC2707010

[51] DerSimonian R , Laird N . Meta‐analysis in clinical trials revisited. Contemp Clin Trials. 2015;45(Pt A):139‐145. 10.1016/j.cct.2015.09.002 26343745 PMC4639420

[52] Higgins JPT , Thompson SG , Deeks JJ , Altman DG . Measuring inconsistency in meta‐analyses. BMJ. 2003;327(7414):557‐560. 10.1136/bmj.327.7414.557 12958120 PMC192859

[53] Egger M , Davey Smith G , Schneider M , Minder C . Bias in meta‐analysis detected by a simple, graphical test. BMJ. 1997;315(7109):629‐634. 10.1136/bmj.315.7109.629 9310563 PMC2127453

[54] Duval S , Tweedie R . Trim and fill: a simple funnel‐plot‐based method of testing and adjusting for publication bias in meta‐analysis. Biometrics. 2000;56(2):455‐463. 10.1111/j.0006-341x.2000.00455.x 10877304

[55] Borenstein M , Hedges L , Higgins JPT , Rothstein HR . Comprehensive Meta‐Analysis (Version 2.2.027). [Computer software]. Biostat; 2005;11:188‐191.

[56] Ducasse D , Jaussent I , Guillaume S , et al. Increased risk of suicide attempt in bipolar patients with severe tobacco dependence. J Affect Disord. 2015;183:113‐118. 10.1016/j.jad.2015.04.038 26001671

[57] Oh KY , Van Dam NT , Doucette JT , Murrough JW . Effects of chronic physical disease and systemic inflammation on suicide risk in patients with depression: a hospital‐based case‐control study. Psychol Med. 2020;50(1):29‐37. 10.1017/S0033291718003902 30606276

[58] Ventorp F , Gustafsson A , Träskman‐Bendz L , Westrin Å , Ljunggren L . Increased soluble urokinase‐type plasminogen activator receptor (suPAR) levels in plasma of suicide attempters. PLoS One. 2015;10(10):e0140052. 10.1371/journal.pone.0140052 26451727 PMC4599802

[59] Gambi F , De Berardis D , Campanella D , et al. A retrospective evaluation of the inflammatory marker C‐reactive protein (CRP), cholesterol and high‐density lipoproteins in patients with major depression: preliminary findings. Eur J Inflamm. 2005;3(3):127‐134. 10.1177/1721727X0500300304

[60] da Graça Cantarelli M , Nardin P , Buffon A , et al. Serum triglycerides, but not cholesterol or leptin, are decreased in suicide attempters with mood disorders. J Affect Disord. 2015;172:403‐409. 10.1016/j.jad.2014.10.033 25451444

[61] De Berardis D , Serroni N , Campanella D , et al. C‐Reactive protein levels and its relationships with suicide risk and alexithymia among newly diagnosed, drug‐naive patients with non affective Psychosis. Int Clin Psychopharmacol. 2012;28:e35. 10.1097/01.yic.0000423298.08154.76

[62] Aguglia A , Solano P , Giacomini G , et al. The association between dyslipidemia and lethality of suicide attempts: a case‐control study. Front Psychiatry. 2019;10:70. 10.3389/fpsyt.2019.00070 30881317 PMC6405629

[63] Aguglia A , Solano P , Parisi VM , et al. Predictors of relapse in high lethality suicide attempters: a six‐month prospective study. J Affect Disord. 2020;271:328‐335. 10.1016/j.jad.2020.04.006 32479332

[64] Dowlati Y , Herrmann N , Swardfager W , et al. A meta‐analysis of cytokines in major depression. Biol Psychiatry. 2010;67(5):446‐457. 10.1016/j.biopsych.2009.09.033 20015486

[65] Valkanova V , Ebmeier KP , Allan CL . CRP, IL‐6 and depression: a systematic review and meta‐analysis of longitudinal studies. J Affect Disord. 2013;150(3):736‐744. 10.1016/j.jad.2013.06.004 23870425

[66] Momtazmanesh S , Zare‐Shahabadi A , Rezaei N . Cytokine alterations in schizophrenia: an updated review. Front Psychiatry. 2019;10:892. 10.3389/fpsyt.2019.00892 31908647 PMC6915198

[67] Yuan N , Chen Y , Xia Y , Dai J , Liu C . Inflammation‐related biomarkers in major psychiatric disorders: a cross‐disorder assessment of reproducibility and specificity in 43 meta‐analyses. Transl Psychiat. 2019;9(1):1‐13. 10.1038/s41398-019-0570-y PMC6751188 31534116

[68] Trovão N , Prata J , VonDoellinger O , Santos S , Barbosa M , Coelho R . Peripheral biomarkers for first‐episode psychosis—opportunities from the neuroinflammatory hypothesis of schizophrenia. Psychiatry Investig. 2019;16(3):177‐184. 10.30773/pi.2018.12.19.1 PMC6444098 30836740

[69] Potvin S , Stip E , Sepehry AA , Gendron A , Bah R , Kouassi E . Inflammatory cytokine alterations in schizophrenia: a systematic quantitative review. Biol Psychiatry. 2008;63(8):801‐808. 10.1016/j.biopsych.2007.09.024 18005941

[70] Modabbernia A , Taslimi S , Brietzke E , Ashrafi M . Cytokine alterations in bipolar disorder: a meta‐analysis of 30 studies. Biol Psychiatry. 2013;74(1):15‐25. 10.1016/j.biopsych.2013.01.007 23419545

[71] Munkholm K , Braüner JV , Kessing LV , Vinberg M . Cytokines in bipolar disorder vs. healthy control subjects: a systematic review and meta‐analysis. J Psychiatr Res. 2013;47(9):1119‐1133. 10.1016/j.jpsychires.2013.05.018 23768870

[72] Dargél AA , Godin O , Kapczinski F , Kupfer DJ , Leboyer M . C‐reactive protein alterations in bipolar disorder: a meta‐analysis. J Clin Psychiatry. 2015;76(2):142‐150. 10.4088/JCP.14r09007 25742201

[73] Steiner J , Bielau H , Brisch R , et al. Immunological aspects in the neurobiology of suicide: elevated microglial density in schizophrenia and depression is associated with suicide. J Psychiatr Res. 2008;42(2):151‐157. 10.1016/j.jpsychires.2006.10.013 17174336

[74] Pandey GN , Rizavi HS , Ren X , et al. Proinflammatory cytokines in the prefrontal cortex of teenage suicide victims. J Psychiatr Res. 2012;46(1):57‐63. 10.1016/j.jpsychires.2011.08.006 21906753 PMC3224201

[75] Tonelli LH , Stiller J , Rujescu D , et al. Elevated cytokine expression in the orbitofrontal cortex of victims of suicide. Acta Psychiatr Scand. 2008;117(3):198‐206. 10.1111/j.1600-0447.2007.01128.x 18081924 PMC2612100

[76] Torres‐Platas SG , Cruceanu C , Chen GG , Turecki G , Mechawar N . Evidence for increased microglial priming and macrophage recruitment in the dorsal anterior cingulate white matter of depressed suicides. Brain Behav Immun. 2014;42:50‐59. 10.1016/j.bbi.2014.05.007 24858659

[77] Schnieder TP , Trencevska I , Rosoklija G , et al. Microglia of prefrontal white matter in suicide. J Neuropathol Exp Neurol. 2014;73(9):880‐890. 10.1097/NEN.0000000000000107 25101704 PMC4141011

[78] Serafini G , Parisi VM , Aguglia A , et al. A specific inflammatory profile underlying suicide risk? Systematic review of the main literature findings. Int J Environ Res Public Health. 2020;17(7):2393. 10.3390/ijerph17072393 PMC7177217 32244611

[79] Goodwin RD , Eaton WW . Asthma, suicidal ideation, and suicide attempts: findings from the Baltimore epidemiologic catchment area follow‐up. Am J Public Health. 2005;95(4):717‐722. 10.2105/AJPH.2003.019109 15798135 PMC1449246

[80] Postolache TT , Stiller JW , Herrell R , et al. Tree pollen peaks are associated with increased nonviolent suicide in women. Mol Psychiatry. 2005;10(3):232‐235. 10.1038/sj.mp.4001620 15599378 PMC7100718

[81] Timonen M , Viilo K , Hakko H , et al. Is seasonality of suicides stronger in victims with hospital‐treated atopic disorders? Psychiatry Res. 2004;126(2):167‐175. 10.1016/j.psychres.2004.02.005 15123396

[82] Dieperink E , Ho SB , Tetrick L , Thuras P , Dua K , Willenbring ML . Suicidal ideation during interferon‐alpha2b and ribavirin treatment of patients with chronic hepatitis C. Gen Hosp Psychiatry. 2004;26(3):237‐240. 10.1016/j.genhosppsych.2004.01.003 15121353

[83] Fragoso YD , Frota ERC , Lopes JS , et al. Severe depression, suicide attempts, and ideation during the use of interferon beta by patients with multiple sclerosis. Clin Neuropharmacol. 2010;33(6):312‐316. 10.1097/WNF.0b013e3181f8d513 21079457

[84] Grunebaum MF , Ramsay SR , Galfalvy HC , et al. Correlates of suicide attempt history in bipolar disorder: a stress‐diathesis perspective. Bipolar Disord. 2006;8(5 Pt 2):551‐557. 10.1111/j.1399-5618.2006.00304.x 17042828

[85] Haapakoski R , Mathieu J , Ebmeier KP , Alenius H , Kivimäki M . Cumulative meta‐analysis of interleukins 6 and 1β, tumour necrosis factor α and C‐reactive protein in patients with major depressive disorder. Brain Behav Immun. 2015;49:206‐215. 10.1016/j.bbi.2015.06.001 26065825 PMC4566946

[86] Wium‐Andersen MK , Ørsted DD , Nielsen SF , Nordestgaard BG . Elevated C‐reactive protein levels, psychological distress, and depression in 73, 131 individuals. JAMA Psychiatry. 2013;70(2):176‐184. 10.1001/2013.jamapsychiatry.102 23266538

[87] Mechawar N , Savitz J . Neuropathology of mood disorders: do we see the stigmata of inflammation? Transl Psychiatry. 2016;6(11):e946. 10.1038/tp.2016.212 27824355 PMC5314124

[88] Rajkowska G , Legutko B , Moulana M , et al. Astrocyte pathology in the ventral prefrontal white matter in depression. J Psychiatr Res. 2018;102:150‐158. 10.1016/j.jpsychires.2018.04.005 29660602 PMC6005746

[89] Maes M , Moraes JB , Bonifacio KL , et al. Towards a new model and classification of mood disorders based on risk resilience, neuro‐affective toxicity, staging, and phenome features using the nomothetic network psychiatry approach. Metab Brain Dis. 2021;36(3):509‐521. 10.1007/s11011-020-00656-6 33411213

[90] Serafini G , Pompili M , Elena Seretti M , et al. The role of inflammatory cytokines in suicidal behavior: a systematic review. Eur Neuropsychopharmacol. 2013;23(12):1672‐1686. 10.1016/j.euroneuro.2013.06.002 23896009

[91] Spampinato SF , Copani A , Nicoletti F , Sortino MA , Caraci F . Metabotropic glutamate receptors in glial cells: a new potential target for neuroprotection? Front Mol Neurosci. 2018;11:414. 10.3389/fnmol.2018.00414 30483053 PMC6243036

[92] Olmos G , Lladó J . Tumor necrosis factor alpha: a link between neuroinflammation and excitotoxicity. Mediators Inflamm. 2014;2014:861231. 10.1155/2014/861231 24966471 PMC4055424

[93] McFadyen JD , Zeller J , Potempa LA , Pietersz GA , Eisenhardt SU , Peter K . C‐reactive protein and its structural isoforms: an evolutionary conserved marker and central player in inflammatory diseases and beyond. Subcell Biochem. 2020;94:499‐520. 10.1007/978-3-030-41769-7_20 32189313

[94] De Berardis D , Serroni N , Campanella D , et al. Alexithymia, suicide ideation, C‐reactive protein, and serum lipid levels among outpatients with generalized anxiety disorder. Arch Suicide Res. 2017;21(1):100‐112. 10.1080/13811118.2015.1004485 25856390

[95] Gan Z , Wu X , Liao Y , et al. The association between low‐grade inflammation and the clinical features of bipolar disorder in Han Chinese population. Psychoneuroendocrinology. 2019;101:286‐294. 10.1016/j.psyneuen.2018.12.239 30597323

[96] Miller BJ , Buckley P , Seabolt W , Mellor A , Kirkpatrick B . Meta‐analysis of cytokine alterations in schizophrenia: clinical status and antipsychotic effects. Biol Psychiatry. 2011;70(7):663‐671. 10.1016/j.biopsych.2011.04.013 21641581 PMC4071300

[97] Miller BJ , Culpepper N , Rapaport MH . C‐reactive protein levels in schizophrenia: a review and meta‐analysis. Clin Schizophr Relat Psychoses. 2014;7(4):223‐230. 10.3371/CSRP.MICU.020813 23428789

[98] Zhang Q , Hong WU , Li H , et al. Increased ratio of high sensitivity C‐reactive protein to interleukin‐10 as a potential peripheral biomarker of schizophrenia and aggression. Int J Psychophysiol. 2017;114:9‐15. 10.1016/j.ijpsycho.2017.02.001 28174109

[99] Orsolini L , Sarchione F , Vellante F , et al. Protein‐C reactive as biomarker predictor of schizophrenia phases of illness? A Systematic Review. Curr Neuropharmacol. 2018;16(5):583‐606. 10.2174/1570159X16666180119144538 29357805 PMC5997872

[100] Barzilay R , Lobel T , Krivoy A , Shlosberg D , Weizman A , Katz N . Elevated C‐reactive protein levels in schizophrenia inpatients is associated with aggressive behavior. Eur Psychiatry. 2016;31:8‐12. 10.1016/j.eurpsy.2015.09.461 26657596

[101] Maes M , Moraes JB , Congio A , et al. Development of a novel staging model for affective disorders using partial least squares bootstrapping: effects of lipid‐associated antioxidant defenses and neuro‐oxidative stress. Mol Neurobiol. 2019;56(9):6626‐6644. 10.1007/s12035-019-1552-z 30911933

[102] Kim S‐Y , Jeon S‐W , Lim W‐J , et al. The relationship between serum vitamin D levels, C‐reactive protein, and anxiety symptoms. Psychiatry Investig. 2020;17(4):312‐319. 10.30773/pi.2019.0290 PMC7176560 32213801

[103] Landry A , Docherty P , Ouellette S , Cartier LJ . Causes and outcomes of markedly elevated C‐reactive protein levels. Can Fam Physician. 2017;63(6):e316‐e323. 28615410 PMC5471098

[104] Coelho R , Viola TW , Walss‐Bass C , Brietzke E , Grassi‐Oliveira R . Childhood maltreatment and inflammatory markers: a systematic review. Acta Psychiatr Scand. 2014;129(3):180‐192. 10.1111/acps.12217 24205846

[105] Smidowicz A , Regula J . Effect of nutritional status and dietary patterns on human serum C‐reactive protein and interleukin‐6 concentrations12. Adv Nutr. 2015;6(6):738‐747. 10.3945/an.115.009415 26567198 PMC4642421

[106] Moraes JB , Maes M , Barbosa DS , et al. Elevated C‐reactive protein levels in women with bipolar disorder may be explained by a history of childhood trauma, especially sexual abuse, body mass index and age. CNS Neurol Disord Drug Targets. 2017;16(4):514‐521. 10.2174/1871527316666170407151514 28403800

[107] Hannestad J , DellaGioia N , Bloch M . The effect of antidepressant medication treatment on serum levels of inflammatory cytokines: a meta‐analysis. Neuropsychopharmacology. 2011;36(12):2452‐2459. 10.1038/npp.2011.132 21796103 PMC3194072

[108] Chang HH , Lee IH , Gean PW , et al. Treatment response and cognitive impairment in major depression: association with C‐reactive protein. Brain Behav Immun. 2012;26(1):90‐95. 10.1016/j.bbi.2011.07.239 21839826

[109] O’Brien SM , Scott LV , Dinan TG . Antidepressant therapy and C‐reactive protein levels. Br J Psychiatry. 2006;188:449‐452. 10.1192/bjp.bp.105.011015 16648531

[110] Tousoulis D , Drolias A , Antoniades C , et al. Antidepressive treatment as a modulator of inflammatory process in patients with heart failure: effects on proinflammatory cytokines and acute phase protein levels. Int J Cardiol. 2009;134(2):238‐243. 10.1016/j.ijcard.2008.02.013 18579238

[111] Carvalho AF , Solmi M , Sanches M , et al. Evidence‐based umbrella review of 162 peripheral biomarkers for major mental disorders. Transl Psychiatry. 2020;10(1):152. 10.1038/s41398-020-0835-5 32424116 PMC7235270

[112] Fusar‐Poli P , Solmi M , Brondino N , et al. Transdiagnostic psychiatry: a systematic review. World Psychiatry. 2019;18(2):192‐207. 10.1002/wps.20631 31059629 PMC6502428

[113] Solmi M , Bodini L , Cocozza S , et al. Aripiprazole monotherapy as transdiagnostic intervention for the treatment of mental disorders: an umbrella review according to TRANSD criteria. Eur Neuropsychopharmacol. 2020;41:16‐27. 10.1016/j.euroneuro.2020.09.635 33077324

[114] Branchi I , Poggini S , Capuron L , et al. Brain‐immune crosstalk in the treatment of major depressive disorder. Eur Neuropsychopharmacol. 2021;45:89‐107. 10.1016/j.euroneuro.2020.11.016 33386229

[115] Del Matto L , Muscas M , Murru A , et al. Lithium and suicide prevention in mood disorders and in the general population: A systematic review. Neurosci Biobehav Rev. 2020;116:142‐153. 10.1016/j.neubiorev.2020.06.017 32561344

