# Supplementary material for: Increased C‐reactive protein concentration and suicidal behavior in people with psychiatric disorders: A systematic review and meta‐analysis
Source: Acta Psychiatr Scand. 2021 Aug 25;144(6):537–52. doi: 10.1111/acps.13351 (PMC9290832; doi:10.1111/acps.13351)
Supplement: Supplementary file 1 — Supinfo S1 [file ACPS-144-537-s001.docx]

Supplementary material

**Increased C-reactive protein concentration and suicidal behavior in people with psychiatric disorders: a systematic review and meta-analysis**

Alessandro Miola, MD;1 Veronica Dal Porto, MD; 1 Tal Tadmor, MD; 1 Giovanni Croatto, MD;1 Paolo Scocco, MD; 2 Mirko Manchia, MD, PhD;3,4,5 Andre F Carvalho, MD;6,7 Michael Maes, MD;8,9 Eduard Vieta, MD;10 Fabio Sambataro, MD, PhD; 1 Marco Solmi, MD, PhD1

1 Neurosciences Department, University of Padua, Italy

2 ULSS 6 Euganea, Psychiatry Department, Padova, Italy

3 Unit of Psychiatry, Department of Public Health, Clinical and Molecular Medicine, University of Cagliari, Cagliari, Italy4 Unit of Clinical Pharmacology, University Hospital Agency of Cagliari, Cagliari, Italy

5 Unit of Clinical Psychiatry, University Hospital Agency of Cagliari, Cagliari, Italy

6 Deakin University, IMPACT - the Institute for Mental and Physical Health and Clinical Translation, School of Medicine, Barwon Health, Geelong, Australia

7 Department of Psychiatry, University of Toronto, Toronto, Ontario, Canada

8 Department of Psychiatry, Faculty of Medicine, King Chulalongkorn Memorial Hospital, Chulalongkorn University, Bangkok, Thailand. dr.michaelmaes@hotmail.com

9 School of Medicine, IMPACT Strategic Research Centre, Deakin University, PO Box 281, Geelong, VIC, 3220, Australia

10 Bipolar and Depressive Disorders Unit, Hospital Clinic, University of Barcelona, Institute of Neuroscience, IDIBAPS, CIBERSAM

Corresponding author

Marco Solmi – marco.solmi83@gmail.com

Via Giustiniani 5, Padua - +390498213831

Neurosciences Department, University of Padua, Italy

**Index**

eTable 1. PRISMA checklist

eTable 2. MOOSE checklist

eTable 3. The list of excluded studies by full text screening with exclusion reason

**eTable 1. PRISMA checklist**^1^

| **Section/topic** | **#** | **Checklist item** | **Reported on page #** | |
| --- | --- | --- | --- | --- |
| **TITLE** | | | |  |
| Title | 1 | Identify the report as a systematic review, meta-analysis, or both. | 1 | |
| **ABSTRACT** | | | |  |
| Structured summary | 2 | Provide a structured summary including, as applicable: background; objectives; data sources; study eligibility criteria, participants, and interventions; study appraisal and synthesis methods; results; limitations; conclusions and implications of key findings; systematic review registration number. | 2 | |
| **INTRODUCTION** | | | |  |
| Rationale | 3 | Describe the rationale for the review in the context of what is already known. | 3 | |
| Objectives | 4 | Provide an explicit statement of questions being addressed with reference to participants, interventions, comparisons, outcomes, and study design (PICOS). | 3 | |
| **METHODS** | | | |  |
| Protocol and registration | 5 | Indicate if a review protocol exists, if and where it can be accessed (e.g., Web address), and, if available, provide registration information including registration number. | 4 | |
| Eligibility criteria | 6 | Specify study characteristics (e.g., PICOS, length of follow-up) and report characteristics (e.g., years considered, language, publication status) used as criteria for eligibility, giving rationale. | 4 | |
| Information sources | 7 | Describe all information sources (e.g., databases with dates of coverage, contact with study authors to identify additional studies) in the search and date last searched. | 4 | |
| Search | 8 | Present full electronic search strategy for at least one database, including any limits used, such that it could be repeated. | 4 | |
| Study selection | 9 | State the process for selecting studies (i.e., screening, eligibility, included in systematic review, and, if applicable, included in the meta-analysis). | 4 | |
| Data collection process | 10 | Describe method of data extraction from reports (e.g., piloted forms, independently, in duplicate) and any processes for obtaining and confirming data from investigators. | 5 | |
| Data items | 11 | List and define all variables for which data were sought (e.g., PICOS, funding sources) and any assumptions and simplifications made. | 5 | |
| Risk of bias in individual studies | 12 | Describe methods used for assessing risk of bias of individual studies (including specification of whether this was done at the study or outcome level), and how this information is to be used in any data synthesis. | 5 | |
| Summary measures | 13 | State the principal summary measures (e.g., risk ratio, difference in means). | 5 | |
| Synthesis of results | 14 | Describe the methods of handling data and combining results of studies, if done, including measures of consistency (e.g., I^2^) for each meta-analysis. | 5 | |
| Risk of bias across studies | 15 | Specify any assessment of risk of bias that may affect the cumulative evidence (e.g., publication bias, selective reporting within studies). | 5 | |
| Additional analyses | 16 | Describe methods of additional analyses (e.g., sensitivity or subgroup analyses, meta-regression), if done, indicating which were pre-specified. | 5 | |
| **RESULTS** | | |  | |
| Study selection | 17 | Give numbers of studies screened, assessed for eligibility, and included in the review, with reasons for exclusions at each stage, ideally with a flow diagram. | 6 | |
| Study characteristics | 18 | For each study, present characteristics for which data were extracted (e.g., study size, PICOS, follow-up period) and provide the citations. | 6, table 1 | |
| Risk of bias within studies | 19 | Present data on risk of bias of each study and, if available, any outcome level assessment (see item 12). | 7, table 3 | |
| Results of individual studies | 20 | For all outcomes considered (benefits or harms), present, for each study: (a) simple summary data for each intervention group (b) effect estimates and confidence intervals, ideally with a forest plot. | Table 3 | |
| Synthesis of results | 21 | Present results of each meta-analysis done, including confidence intervals and measures of consistency. | 6, table 3 | |
| Risk of bias across studies | 22 | Present results of any assessment of risk of bias across studies (see Item 15). | 7, table 3 | |
| Additional analysis | 23 | Give results of additional analyses, if done (e.g., sensitivity or subgroup analyses, meta-regression [see Item 16]). | 7, table 3 | |
| **DISCUSSION** | | |  | |
| Summary of evidence | 24 | Summarize the main findings including the strength of evidence for each main outcome; consider their relevance to key groups (e.g., healthcare providers, users, and policy makers). | 7, 8 | |
| Limitations | 25 | Discuss limitations at study and outcome level (e.g., risk of bias), and at review-level (e.g., incomplete retrieval of identified research, reporting bias). | 9, 10 | |
| Conclusions | 26 | Provide a general interpretation of the results in the context of other evidence, and implications for future research. | 10 | |
| **FUNDING** | | |  | |
| Funding | 27 | Describe sources of funding for the systematic review and other support (e.g., supply of data); role of funders for the systematic review. | 10 | |

*From:*  Moher D, Liberati A, Tetzlaff J, Altman DG, The PRISMA Group (2009). Preferred Reporting Items for Systematic Reviews and Meta-Analyses: The PRISMA Statement. PLoS Med 6(7): e1000097. doi:10.1371/journal.pmed1000097

eTable 2. MOOSE Checklist for Meta-analyses of Observational Studies^2^

| **Item No** | **Recommendation** | **Reported**  **on Page No** |
| --- | --- | --- |
| Reporting of background should include | | |
| 1 | Problem definition | 3, 4 |
| 2 | Hypothesis statement | 3, 4 |
| 3 | Description of study outcome(s) | 4 |
| 4 | Type of exposure or intervention used | 4 |
| 5 | Type of study designs used | 4 |
| 6 | Study population | 4 |
| Reporting of search strategy should include | | |
| 7 | Qualifications of searchers (eg, librarians and investigators) | 4 |
| 8 | Search strategy, including time period included in the synthesis and key words | 4 |
| 9 | Effort to include all available studies, including contact with authors | 4 |
| 10 | Databases and registries searched | 4 |
| 11 | Search software used, name and version, including special features used (eg, explosion) | 4 |
| 12 | Use of hand searching (eg, reference lists of obtained articles) | 4 |
| 13 | List of citations located and those excluded, including justification | 5, Table 2, eTable 3 |
| 14 | Method of addressing articles published in languages other than English | NA |
| 15 | Method of handling abstracts and unpublished studies | 5 |
| 16 | Description of any contact with authors | NA |
| Reporting of methods should include | | |
| 17 | Description of relevance or appropriateness of studies assembled for assessing the hypothesis to be tested | 5 |
| 18 | Rationale for the selection and coding of data (eg, sound clinical principles or convenience) | 5 |
| 19 | Documentation of how data were classified and coded (eg, multiple raters, blinding and interrater reliability) | 5 |
| 20 | Assessment of confounding (eg, comparability of cases and controls in studies where appropriate) | 5 |
| 21 | Assessment of study quality, including blinding of quality assessors, stratification or regression on possible predictors of study results | 5 |
| 22 | Assessment of heterogeneity | 5 |
| 23 | Description of statistical methods (eg, complete description of fixed or random effects models, justification of whether the chosen models account for predictors of study results, dose-response models, or cumulative meta-analysis) in sufficient detail to be replicated | 5, Table 1 |
| 24 | Provision of appropriate tables and graphics | Table 1, 2, 3, 4 Figure 1, eTable 1, 2, 3, |
| Reporting of results should include | | |
| 25 | Graphic summarizing individual study estimates and overall estimate | NA |
| 26 | Table giving descriptive information for each study included | Table 1 |
| 27 | Results of sensitivity testing (eg, subgroup analysis) | Table 3,4 |
| 28 | Indication of statistical uncertainty of findings | 6, Table 2,3,4 |

| **Item No** | **Recommendation** | **Reported**  **on Page No** |
| --- | --- | --- |
| Reporting of discussion should include | | |
| 29 | Quantitative assessment of bias (eg, publication bias) | 7, Table 3,4 |
| 30 | Justification for exclusion (eg, exclusion of non-English language citations) | eTable 3 |
| 31 | Assessment of quality of included studies | 6, Table 2 |
| Reporting of conclusions should include | | |
| 32 | Consideration of alternative explanations for observed results | 9, 10 |
| 33 | Generalization of the conclusions (ie, appropriate for the data presented and within the domain of the literature review) | 9, 10 |
| 34 | Guidelines for future research | 10 |
| 35 | Disclosure of funding source | 10 |

**eTable 3. List of studies excluded after full-text assessment, with reason for exclusion.**

| **Author, year** | **Reason for exclusion** |
| --- | --- |
| Batty, 2016 ^3^ | General population |
| Caceda, 2018 ^4^ | No control group |
| Chang 2018 ^5^ | No data |
| Coryell, 2020 ^6^ | No data |
| De Berardis 2015 ^7^ | No data |
| De Berardis, 2008 ^8^ | No control group |
| Gan, 2019 ^9^ | No data |
| Goldstein, 2015 ^10^ | No data |
| Khandaker, 2014 ^11^ | No data |
| Köhler-Forsberg, 2017 ^12^ | No data |
| Lindgrem, 2020 ^13^ | No data |
| Maes, 2018 ^14^ | No data |
| Melhem, 2017 ^15^ | No data |
| O' Donovan, 2010 ^16^ | No data |
| Park, 2017 ^17^ | General population |
| Russell, 2019 ^18^ | No data |
| Russell, 2020 ^19^ | No data |
| Shim, 2016 ^20^ | No data |
| Suchankova, 2013 ^21^ | No data |

**Supplementary references**

1. Moher D, Liberati A, Tetzlaff J, Altman DG. Preferred reporting items for systematic reviews and meta-analyses: the PRISMA statement. *J Clin Epidemiol*. 2009;62(10):1006-1012.

2. Stroup DF, Berlin JA, Morton SC, et al. Meta-analysis of observational studies in epidemiology: A proposal for reporting. *J Am Med Assoc*. 2000;283(15):2008-2012.

3. Batty GD, Bell S, Stamatakis E, Kivimäki M. Association of Systemic Inflammation With Risk of Completed Suicide in the General Population. *JAMA Psychiatry*. 2016;73(9):993-995.

4. Cáceda R, Griffin WST, Delgado PL. A probe in the connection between inflammation, cognition and suicide. *J Psychopharmacol Oxf Engl*. 2018;32(4):482-488.

5. Chang HB, Munroe S, Gray K, et al. The role of substance use, smoking, and inflammation in risk for suicidal behavior. *J Affect Disord*. 2019;243:33-41.

6. Coryell W, Wilcox H, Evans SJ, et al. Latent infection, inflammatory markers and suicide attempt history in depressive disorders. *J Affect Disord*. 2020;270:97-101.

7. De Berardis D, Serroni N, Campanella D, et al. Alexithymia, Suicide Ideation, C-Reactive Protein, and Serum Lipid Levels Among Outpatients with Generalized Anxiety Disorder. *Arch Suicide Res Off J Int Acad Suicide Res*. 2017;21(1):100-112.

8. De Berardis D, Serroni N, Campanella D, et al. Alexithymia and its relationships with C-reactive protein and serum lipid levels among drug naïve adult outpatients with major depression. *Prog Neuropsychopharmacol Biol Psychiatry*. 2008;32(8):1982-1986.

9. Gan Z, Wu X, Liao Y, et al. The association between low-grade inflammation and the clinical features of bipolar disorder in Han Chinese population. *Psychoneuroendocrinology*. 2019;101:286-294.

10. Goldstein BI, Lotrich F, Axelson DA, et al. Inflammatory markers among adolescents and young adults with bipolar spectrum disorders. *J Clin Psychiatry*. 2015;76(11):1556-1563.

11. Khandaker GM, Pearson RM, Zammit S, Lewis G, Jones PB. Association of serum interleukin 6 and C-reactive protein in childhood with depression and psychosis in young adult life: a population-based longitudinal study. *JAMA Psychiatry*. 2014;71(10):1121-1128.

12. Köhler-Forsberg O, Buttenschøn HN, Tansey KE, et al. Association between C-reactive protein (CRP) with depression symptom severity and specific depressive symptoms in major depression. *Brain Behav Immun*. 2017;62:344-350.

13. Lindgren M, Holm M, Markkula N, et al. Exposure to common infections and risk of suicide and self-harm: a longitudinal general population study. *Eur Arch Psychiatry Clin Neurosci*. 2020;270(7):829-839.

14. Maes M, Congio A, Moraes JB, et al. Early Life Trauma Predicts Affective Phenomenology and the Effects are Partly Mediated by Staging Coupled with Lowered Lipid-Associated Antioxidant Defences. *Biomol Concepts*. 2018;9(1):115-130.

15. Melhem NM, Munroe S, Marsland A, et al. Blunted HPA axis activity prior to suicide attempt and increased inflammation in attempters. *Psychoneuroendocrinology*. 2017;77:284-294.

16. O’Donovan A, Hughes BM, Slavich GM, et al. Clinical anxiety, cortisol and interleukin-6: evidence for specificity in emotion-biology relationships. *Brain Behav Immun*. 2010;24(7):1074-1077.

17. Park RJ, Kim YH. Association between high sensitivity CRP and suicidal ideation in the Korean general population. *Eur Neuropsychopharmacol J Eur Coll Neuropsychopharmacol*. 2017;27(9):885-891.

18. Russell AE, Heron J, Gunnell D, et al. Pathways between early-life adversity and adolescent self-harm: the mediating role of inflammation in the Avon Longitudinal Study of Parents and Children. *J Child Psychol Psychiatry*. 2019;60(10):1094-1103.

19. Russell AE, Ford T, Gunnell D, et al. Investigating evidence for a causal association between inflammation and self-harm: A multivariable Mendelian Randomisation study. *Brain Behav Immun*. 2020;89:43-50.

20. Shim IH, Woo YS, Bahk W-M. Associations between immune activation and the current severity of the “with anxious distress” specifier in patients with depressive disorders. *Gen Hosp Psychiatry*. 2016;42:27-31.

21. Suchankova P, Holm G, Träskman-Bendz L, Brundin L, Ekman A. The +1444C>T polymorphism in the CRP gene: a study on personality traits and suicidal behaviour. *Psychiatr Genet*. 2013;23(2):70-76.
